# Development and Characterization of Andrographolide Microparticles via Spray Drying: An Aqueous-Based Chitosan/Cellulose/Poloxamer Carrier Approach

**DOI:** 10.3390/polym18131655

**Published:** 2026-07-03

**Authors:** Nuttapong Khiaonoi, Kwanchai Kraitong, Punyawan Lumpaopong, Jarupa Viyoch

**Affiliations:** 1 Department of Mechanical Engineering, Faculty of Engineering, Naresuan University, Phitsanulok 65000, Thailand; nuttapongk65@nu.ac.th (N.K.); kwanchaik@nu.ac.th (K.K.); 2 Department of Pharmaceutical Technology, Faculty of Pharmaceutical Sciences, Naresuan University, Phitsanulok 65000, Thailand; 3 Center of Excellence for Innovation in Chemistry (PERCH-CIC), Faculty of Pharmaceutical Sciences, Naresuan University, Phitsanulok 65000, Thailand

**Keywords:** chitosan/cellulose/poloxamer, andrographolide, spray-dried microparticles, pulmonary drug delivery

## Abstract

Andrographolide-loaded microparticles with an aqueous-based carrier system were developed with the aim of pulmonary drug delivery. Five formulations of andrographolide (0.6–5.8% *w*/*w*) loaded on mixed-polymer carriers containing chitosan (CHS), hydroxyethyl cellulose (HEC), Poloxamer 188, and PEG 20,000, with various ratios were produced under various spray-drying parameters: solution viscosity (5–20 cP), atomization air pressure (0.8–1.5 bar) and solution feed rate (3–6 mL/min). The physiochemical properties of the microparticles were strongly affected by carrier composition and atomization air pressure. The optimal formulation: andrographolide 0.6% *w*/*w*, CHS 62.2% *w*/*w*, HEC 15.5% *w*/*w* and Poloxamer 188 21.7% *w*/*w*, spray dried using solution viscosity 15 cP, atomization air pressure 1.1 bar and feed rate 3 mL/min, was selected according to its particle sizes (3–5 µm) with rough morphology, encapsulation efficiency (54.47%) and release behaviors (22.31%/h and 89.23% within 4 h). Good physical, chemical, and thermal stabilities under room storage condition (28 ± 2 °C, 50% relative humidity) were also proven. Importantly, it demonstrated potent antiviral activity against Influenza A/H1N1, achieving a 3.3-log_10_ reduction in viral titer with 99.95% inhibition. Overall, this aqueous-based carrier approach and spray-drying technique offer a stable and effective inhalable formulation for localized treatment of influenza infections.

## 1. Introduction

Current antiviral agents such as oseltamivir, peramivir, and zanamivir exhibit therapeutic limitations including drug resistance and adverse effects. Consequently, research has shifted toward natural compounds with antiviral activity and a favorable safety profile [1,2,3]. Among natural sources with antiviral properties, andrographis paniculata is well recognized for its effectiveness in treating influenza; when used alongside conventional medications, it can reduce fever duration and minimize the risk of complications [4,5]. This plant contains various bioactive diterpenoids, including andrographolide, neoandrographolide, and 14-deoxyandrographolide [6]. Among these compounds, andrographolide exhibits the most potent antiviral activity by blocking hemagglutinin and neuraminidase to inhibit viral entry, release, and replication [7]. Additionally, it demonstrates anti-inflammatory properties by suppressing NF-κB-mediated cytokine production [8]. Previous studies reported that andrographolide suppressed H9N2, H5N1 and H1N1 viruses at concentrations of 7.2–15.2 μM [9] and inhibited H1N1 activity in 16HBE cells by 43.9% at 250 µg/mL [10]. To deliver andrographolide for inhibiting viruses commonly localized in the respiratory tract, dry powder inhalation represents a suitable dosage form as it enables direct pulmonary deposition, achieving high local andrographolide concentrations while minimizing systemic exposure [11]. However, poor aqueous solubility and the instability of andrographolide lead to low bioavailability and limited absorption in the lung tissues. The encapsulation of andrographolide in polymeric matrices and subsequent formulation into dry powders provides a promising approach for pulmonary delivery. Polymeric carriers, especially hydrophilic and amphiphilic systems, can improve the dispersion, dissolution in lung lining fluid and permeation through mucus of a drug. As a result, these systems contribute to improved pulmonary transport and epithelial uptake, leading to enhanced therapeutic efficacy of andrographolide [12,13]. Beyond particle size considerations, particle surface characteristics and surface charge constitute fundamental parameters that critically determine the efficacy of dry powder inhalation formulations. Strategic modification of particle surface morphology, specifically surface roughness and porosity, represents a key approach for optimizing aerosolization performance by increasing effective surface area while minimizing interparticle cohesive forces [14]. Importantly, particle surface charge significantly influences pulmonary deposition efficiency through electrostatic interactions with the charged airway mucus layer, directly affecting penetration of active compound, retention, and therapeutic outcomes [15].

Chitosan, cellulose derivatives, and Poloxamer are commonly employed as polymeric carriers for active compounds to optimize their performance [16]. Chitosan protects active compounds from degradation, enhances mucus permeation, improves mucoadhesion, and facilitates opening of epithelial tight junctions to increase absorption [17]. Cellulose derivatives stabilize formulations, facilitate penetration through airway mucus, and enable controlled active compound release while protecting labile compounds [18]. Poloxamer enhances water affinity, solubility, permeability, and overall particle stability [19]. Utilization of these polymers in appropriate ratios combined with spray drying represents an effective approach for producing inhalable particles, allowing precise control over particle size, morphology, and surface characteristics. Furthermore, water-based carrier systems are preferable in terms of safety and environmental considerations, as they enhance safety, reduce costs, and eliminate risks associated with residual organic solvents [20].

Previous studies have shown that polymeric carriers could improve the physicochemical properties of andrographolide. Ma et al. (2018) [21] reported the use of cellulose-based systems to enhance andrographolide stability and provide controlled release. However, the near-neutral to slightly negative surface charge of cellulose enhances mucus penetration but weakens mucoadhesion, thereby limiting epithelial uptake and pulmonary absorption. Zhang et al. (2022) [22] demonstrated that solid dispersion systems can improve the solubility and dissolution of andrographolide through determining a suitable carrier and andrographolide–polymer interactions by comparing various single-polymer systems: andrographolide-PEG 8000, andrographolide–poloxamer 188 and andrographolide–Soluplus^®^. The study investigated oral delivery, not pulmonary transport. Sari et al. (2019) [23] developed a chitosan-based system optimizing solubility; however, the strong mucoadhesive nature of chitosan may hinder diffusion through the mucus layer, thereby limiting pulmonary transport. It is also noted that the preparation methods of these studies were based on organic solvents which may raise issues of solvent residues, scalability and pulmonary safety. Using water-based solvent systems provides advantages in terms of safety and regulatory compliance. Nevertheless, limited research has been conducted on spray-dried andrographolide. Previous work by Chen et al. (2018) [24] primarily targeted oral delivery, with limited evaluation of aerosolization behavior and lung deposition for pulmonary application. Notably, spray-dried andrographolide using aqueous-based solvent systems for inhalation delivery has not yet been reported in the literature.

Pulmonary drug delivery efficacy essentially relies on the ability to produce microparticles with appropriate physicochemical and aerodynamic properties. Achieving an aerodynamic particle size of 3–5 µm, low density, and appropriate surface morphology [25] remains problematic when using water-based systems and single-polymer encapsulation approaches, particularly for spray-dried andrographolide. Powder dispersibility is influenced by interparticle interactions (e.g., van der Waals forces) and mechanical properties (e.g., Young’s modulus and coefficient of restitution), where strong cohesion promotes agglomeration and reduces aerosol performance [26,27]. In addition, biological interactions, including mucosal adhesion and epithelial penetration, affect drug delivery after deposition [28]. Thus, effective formulation requires the integration of both physical and biological design criteria [25,28]. This can be achieved using a mixed-polymer encapsulation system to optimize particle properties, improve dispersibility and mucoadhesion, and to control drug release [29].

To address these challenges, this study aimed to develop spray-dried andrographolide powder using an aqueous system by varying carrier (chitosan/hydroxyethyl cellulose/poloxamer) ratios and spray-drying parameters to achieve particles with optimal inhalation properties. In addition, this work investigated how particle physicochemical characteristics influence andrographolide encapsulation efficiency, release kinetics, and antiviral activity against Influenza A/H1N1. The findings provide valuable insights into the design of water-based polymeric carrier systems for andrographolide microencapsulation, offering a promising approach for effective inhalation dosage forms.

## 2. Materials and Methods

### 2.1. Materials

Andrographolide (purity ≥ 99%) was produced by Chanjao Longevity (Bangkok, Thailand) and used as the active compound. The biopolymers consisted of chitosan (CHS; degree of deacetylation, 90%; molecular weight (MW), 150–400 kDa) supplied by Bio21 (Samutsakhon, Thailand) and hydroxyethyl cellulose (HEC; average MW, 90–250 kDa) supplied by Vejchakit Chemical (Phisanulok, Thailand). Poly(ethylene glycol) 20,000 (PEG 20,000) was supplied by Sigma-Aldrich (St. Louis, MO, USA) and used as a copolymer and solubility agent. Poloxamer 188 (MW, 7.6–9.5 kDa) was produced by Chanjao Longevity and used as a solubility enhancer. Sodium bicarbonate was obtained from Sigma-Aldrich.

### 2.2. Preliminary Studies for Formulation Design

Preliminary spray-drying feasibility screening was conducted to define an acceptable feed-viscosity range. Under the selected spray-drying conditions, polymer-based feed dispersions with viscosity approaching approximately 25 cP tended to produce relatively large microparticles, approximately 10 µm in size. Representative scanning electron microscopy (SEM) images from this preliminary screening are provided in Supplementary Figure S1. Since the desired particle-size range for pulmonary deposition was 3–5 µm [25], the feed viscosity in this study was therefore maintained within the range of 5–20 cP to support stable atomization and the production of microparticles within the desired pulmonary size range.

Poloxamer 188 was selected as an amphiphilic solubilizing polymer to improve andrographolide dispersion in the aqueous feed system. This selection was based on literature reports indicating that Poloxamer 188 provides an appropriate balance of micelle-forming ability, hydrophilic character, and acceptable viscosity for spray-drying applications [19,30]. Its content was then controlled at approximately 20–30% *w*/*w* of the total solid composition in the formulation design. The Poloxamer 188-to-andrographolide proportion was optimized by visual observation of feed homogeneity and visible precipitation after mixing. The selected proportion produced a visually homogeneous dispersion without visible andrographolide precipitation and was used in the final formulation design. The qualitative screening data are provided in Supplementary Table S1.

HEC was selected as the cellulose-based carrier because of its nonionic hydrophilic character and suitability for aqueous dispersion, while avoiding strong electrostatic complexation between anionic Carboxymethyl Cellulose (CMC) and cationic chitosan. Hydroxypropyl Methylcellulose (HPMC) was considered as an alternative cellulose as well. However, it is commonly used as a hydrophilic matrix-forming polymer for sustained or controlled release [31,32,33], whereas the formulation of this study was designed for rapid andrographolide release.

The ratio of CHS to HEC was then optimized to 1:1, 2:1, and 4:1 based on visual assessment of feed homogeneity, visible precipitation, and polymer aggregation after mixing. The 4:1 ratio produced a visually homogeneous dispersion without visible precipitation or polymer aggregation and was selected for the final formulation design. The qualitative screening data are summarized in Supplementary Table S2.

### 2.3. Preparation of Spray-Drying Feed Solution Formulations

Andrographolide and polymer compositions, as well as their concentrations, were selected based on the preliminary studies described above to obtain homogeneous feed dispersions without visible precipitation after mixing and to ensure satisfactory spray-drying performance without nozzle clogging. Poloxamer 188 was dissolved in 10 mL of deionized water at a concentration of 2–4% *w*/*v*. Subsequently, 10.0 mg of andrographolide was incorporated into the Poloxamer 188 dispersion and vortex-mixed at 2000–3000 rpm for 15 min to obtain a homogeneous andrographolide dispersion. Two polymeric carriers with different surface characteristics, CHS and HEC, were prepared separately. CHS was dissolved at a concentration of 0.5% *w*/*v* in a mild organic acid solution (0.5–1%, *v*/*v*), following a previously reported method [34]. HEC was dissolved in deionized water at a concentration of 0.0625% *w*/*v*. For additional formulations, PEG 20,000 was prepared separately as an aqueous dispersion at a concentration of 0.01% *w*/*v* before incorporation into the polymer system. The polymer solutions were then combined and added to the andrographolide dispersion under magnetic stirring at 200–300 rpm for 20–30 min to obtain the final feed dispersion for spray drying. The pH of the final dispersion was adjusted to 4.5–5.5 using sodium bicarbonate. Table 1 summarizes the compositions of the five formulations used in this study.

### 2.4. Spray-Drying Conditions

The spray-drying procedure was carried out using a Büchi Mini Spray Dryer B-290 (Büchi Labortechnik AG, Flawil, Switzerland) under 50% to 60% relative humidity (RH). The viscosity of the feed solution ranged from 5 to 20 cP (Brookfield VD2TLV viscometer, Brookfield Engineering Laboratories, Middleborough, MA, USA). The intake and output air temperatures were set at 120 °C and 90 °C, respectively. The atomization air pressure was adjusted between 0.8 and 1.5 bar. The key process parameters were systematically adjusted in accordance with the formulation design. The impact of these parameters on particle size, surface charge, and surface morphology was evaluated. The resulting powder was collected, weighed, and stored in a desiccator. Based on the preliminary composition-range screening and spray-drying feasibility studies, the corresponding spray-drying conditions for F1–F5 are also shown in Table 1.

### 2.5. Microparticle Characteristics

#### 2.5.1. Morphology and Particle Size

The morphology, average diameter, and size distribution of the spray-dried microparticles were analyzed by SEM (FE-SEM Apreo, Thermo Fisher Scientific, Waltham, MA, USA). The powder samples were first gently dispersed onto conductive adhesive carbon tape mounted on an aluminum stub to minimize agglomeration. The mounted samples were then dried at 40 °C for 12 h to remove residual moisture. Subsequently, the samples were sputter-coated with a thin layer of gold under vacuum to enhance conductivity. SEM observations were performed at magnifications ranging from 1kx to 5kX using an accelerating voltage of 5–10 kV to evaluate particle morphology, surface characteristics, and agglomeration behavior. Thirty particles per formulation were measured to determine the mean particle size and size distribution.

#### 2.5.2. Structure and Characterization of Particles

The structural characteristics of the individual components and the formulations were investigated through X-ray diffraction (XRD, D2 PHASER, Bruker AXS, Karlsruhe, Germany) to assess the crystallization behavior of the andrographolide–polymer matrix. The changes in the unique crystalline peaks of andrographolide were used to evaluate crystallinity, andrographolide dispersion, and the possibility of amorphization inside the polymer matrix [35]. Bulk density, tapped density, and porosity were assessed to analyze powder packing characteristics and internal microparticle structure [36]. Porosity was calculated from the bulk and tapped densities according to the following equation:

$$\mathrm{Porosity}\;\left(\%\right)\;=\;\left[\left(\mathrm{Tapped}\;\mathrm{density}\;-\;\mathrm{Bulk}\;\mathrm{density}\right)/\mathrm{Tapped}\;\mathrm{density}\right]\;\times \;100$$

The porosity data were interpreted together with XRD results to support the evaluation of formulation structure. Elastic properties of the tapped powder bed were measured using a Texture Analyzer (A.XTplus Texture Analyzer, Stable Micro Systems, Godalming, UK) equipped with a 2 mm cylindrical probe. The probe penetrated the powder sample contained in a cylindrical holder (5 mm diameter × 4 mm height) to a depth of 2 mm, while force–displacement data were recorded. Young’s modulus was calculated from the linear elastic region of the stress–strain curve, and the coefficient of restitution (COR) was estimated from the ratio of withdrawal force to maximum penetration force, reflecting elastic recovery and energy dissipation [37]. Mechanical property data are presented as descriptive statistics (mean ± SD, *n* = 3) and were used to support interpretation of particle structure rather than formulation selection. For zeta-potential measurements, microparticles were dispersed in deionized water at a concentration of 0.1 mg/mL and gently mixed to obtain a homogeneous suspension. Measurements were performed at 25 °C using a Zetasizer Nano ZS (Malvern Instruments, Worcestershire, UK) equipped with a folded capillary cell. The surface charges of particles in air were estimated from the measured zeta-potential values based on electrokinetic theory [38]. The estimated values were used to evaluate electrostatic interactions among particles and their stability in dispersion.

### 2.6. Encapsulation Efficiency of Andrographolide

The encapsulation efficiency of andrographolide was determined as the percentage of andrographolide encapsulated in the microparticles with respect to the initial amount added. Formulations F1–F5 were dispersed in methanol at a concentration of 0.1% (*w*/*v*), vortex-mixed (8000 rpm), sonicated for 15 min and centrifuged at 15,000 rpm for 20 min to ensure complete extraction of the andrographolide for encapsulation efficiency analysis. The supernatant was collected and analyzed using high-performance liquid chromatography (HPLC; Shimadzu LC-20, Kyoto, Japan). HPLC was carried out on a system equipped with a photodiode array (PDA) detector and a C18 column (250 mm × 4.6 mm, 5 μm). The mobile phase was acetonitrile and 0.1% orthophosphoric acid (40:60, *v*/*v*) at a flow rate of 1.0 mL/min, an injection volume of 20 μL and a detection wavelength of 226 nm [34]. A calibration curve was constructed using standard andrographolide solutions. The HPLC peak area of each sample was converted into concentration (µg/mL) using the linear regression equation of the calibration curve. The amount of andrographolide was then calculated in milligrams (mg) based on the sample volume. Method sensitivity was evaluated in terms of limit of detection (LOD) and limit of quantification (LOQ).

The validated method was subsequently applied to quantify andrographolide for encapsulation efficiency determination, as shown below:

$$\mathrm{Encapsulation}\;\mathrm{efficiency}\;(\%)=\;\frac{\mathrm{Amount}\;\mathrm{of}\;\mathrm{andrographolide}\;\mathrm{encapsulated}}{\mathrm{Total}\;\mathrm{amount}\;\mathrm{of}\;\mathrm{andrographolide}\;\mathrm{added}}\times 100$$

### 2.7. Release Behavior of Andrographolide

The in vitro release of andrographolide from the microparticles was evaluated following a previously reported method [39]. A 4 h release period was selected to reflect the intended rapid pulmonary drug delivery and mucociliary clearance in the tracheobronchial region [40]. Τhe procedure was as follows: 5 mg of each formulation was dispersed in phosphate-buffered saline (PBS) containing 0.2% Tween 20 and incubated at 37 °C under gentle agitation at 150 rpm. Samples were collected at predetermined time points (1, 2, 3, and 4 h), centrifuged, and the supernatants were analyzed for andrographolide content by HPLC under the same conditions described above. The cumulative andrographolide release was calculated using the following equation:

$$\mathrm{Cumulative}\;\mathrm{release}\;(\%)\;=\;\frac{\mathrm{Amount}\;\mathrm{of}\;\mathrm{andrographolide}\;\mathrm{released}}{\mathrm{Total}\;\mathrm{amount}\;\mathrm{of}\;\mathrm{andrographolide}\;\mathrm{encapsulated}}\times 100$$

### 2.8. Physicochemical Characterization of the Optimal Formulation

The optimal formulation was selected based on the following criteria:

1. Particle size between 3 and 5 µm to ensure effective pulmonary deposition [25].
2. Rough particle surface morphology with a near-neutral surface charge to facilitate microparticle deposition at the mucus membrane [14,15].
3. COR > 0.5 for good mechanical resilience [27].
4. Encapsulation efficiency and andrographolide release were considered key criteria. A minimum encapsulation efficiency (≥30%) was set for preliminary screening to ensure adequate drug loading, while a cumulative release of ≥50% within 4 h was targeted to enable rapid drug availability following pulmonary deposition, consistent with the fast–acting nature of inhalation systems [41].

The physicochemical parameters of the chosen formulation were investigated using Fourier-transform infrared spectroscopy (FTIR, PerkinElmer, Waltham, MA, USA), differential scanning calorimetry (DSC, Mettler–Toledo, Greifensee, Switzerland), and thermogravimetric analysis (TGA, GA 4000, PerkinElmer, Waltham, MA, USA) to assess intermolecular interactions and thermal behaviors.

### 2.9. Stability Study of the Optimal Formulation

Two aliquots of the optimal formulation were removed from the bulk and stored under different conditions: (1) at room temperature (28 ± 2 °C, 50% relative humidity (RH)) for 3 months and (2) under accelerated thermal stress conditions (50 ± 2 °C, 70% RH) [42] for 1 month. The andrographolide content was quantified using a validated HPLC method, while physicochemical stability was assessed through XRD and DSC to investigate changes in crystallinity and thermal properties.

### 2.10. Biological Activity of the Optimal Formulation

The optimal formulation was evaluated for antiviral activity against Influenza A/H1N1 (A/Puerto Rico/8/1934, ATCC VR-95) following ASTM E1053-20 guidelines [43] in a biosafety laboratory approved under protocol number NUIBC-MI-67-11-66. The antiviral assay was performed by an independent third-party testing laboratory accredited to ISO/IEC 17025 standards [44] for testing and calibration laboratories. Samples of placebo particles and particles containing andrographolide (300 µg/mL) were exposed to the virus for 60 min. The residual infectivity was quantified as TCID_50_ per 100 µL to determine antiviral efficacy according to U.S. EPA OCSPP 810.2200 criteria [43].

## 3. Results

### 3.1. Spray-Drying Conditions and Formulation Composition

Five formulations (F1–F5) of andrographolide-loaded microparticles were successfully prepared by spray drying using various formulation ratios and spray-drying parameters. The correlations between feed solution viscosity and atomizing air pressure with the resulting microparticle characteristics are described in Table 2.

### 3.2. Surface Morphology and Particle Size of Formulations

**Figure 1:**
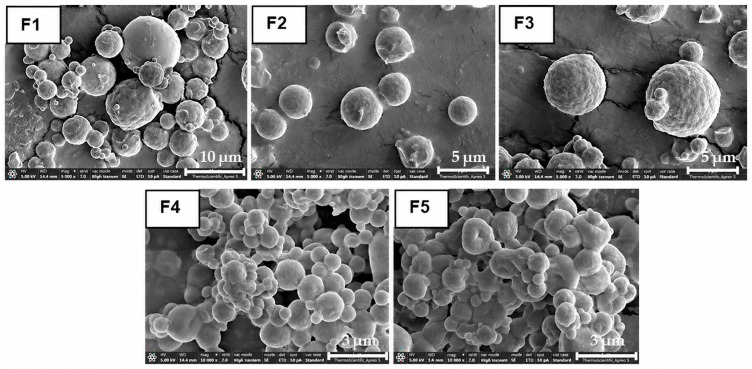
Representative SEM images of andrographolide-loaded microparticles prepared from five formulations. Images were recorded at an accelerating voltage of 5 kV and magnification factor of 5kX.

**Figure 2:**
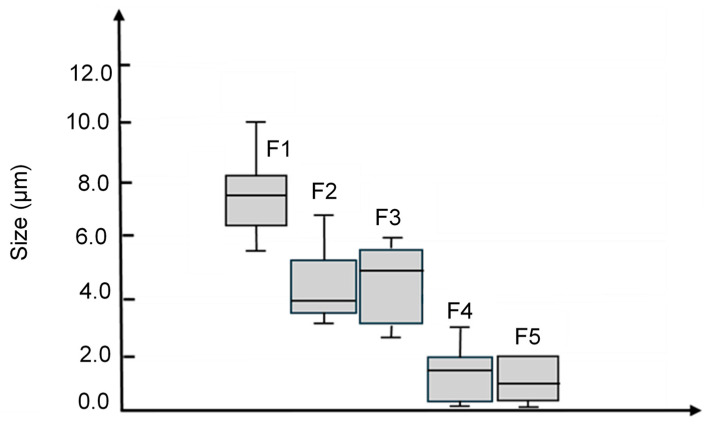
Particle size distribution of spray-dried microparticle formulations.

An analysis using SEM found variations in surface morphology and particle sizes across the spray-dried formulations. Considering the same polymer compositions of formulations F1–F3, varying feed solution viscosity and atomizing air pressure resulted in variations in particle size with similar surface morphology. In contrast, formulations F4 and F5, produced at higher atomizing air pressures, formed particles typically under 3 µm with smoother surfaces, as shown in Figure 1. The span of particle sizes of all formulations under various spray-drying conditions is displayed in Figure 2.

### 3.3. Structural and Mechanical Properties

**Figure 3:**
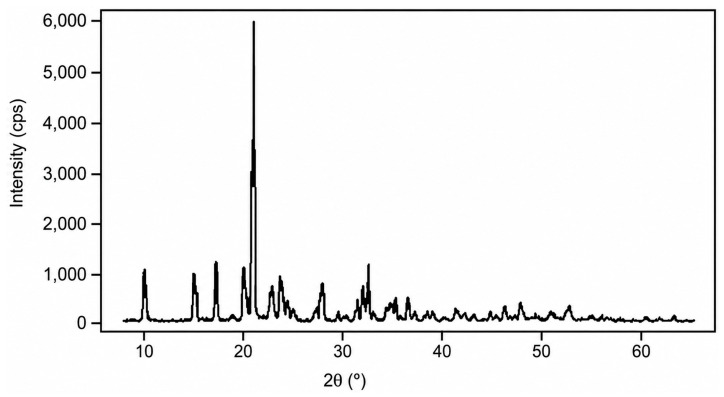
XRD pattern of andrographolide.

**Figure 4:**
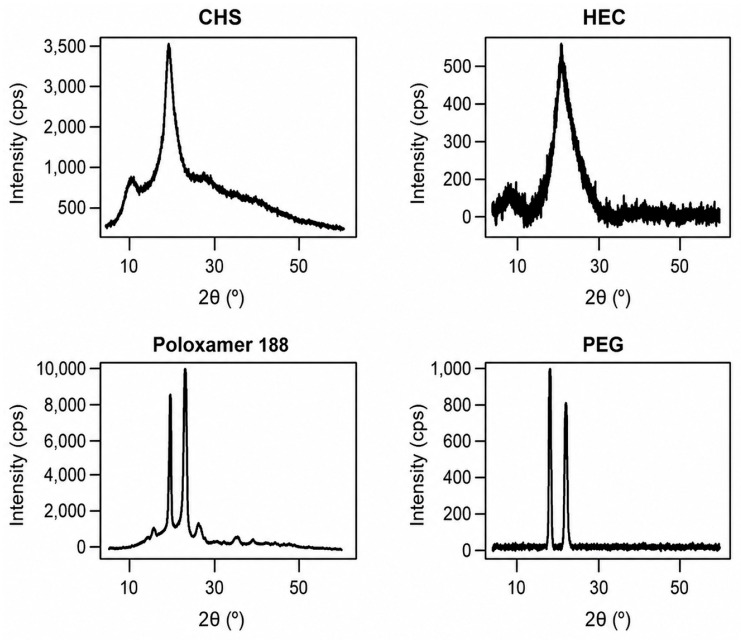
XRD patterns of polymer components including chitosan (CHS; degree of deacetylation, 90%; MW, 150–400 kDa), hydroxyethyl cellulose (HEC; average MW, 90–250 kDa, Poloxamer 188 (MW, 7.6–9.5 kDa), and poly(ethylene glycol) 20,000 (PEG 20,000).

The XRD diffraction profile of andrographolide is shown in Figure 3, whereas the profiles of the polymeric components are displayed in Figure 4. These measurements were used as baseline references for assessing the microparticle structure of each formulation. As shown in Figure 3, andrographolide exhibits multiple sharp diffraction peaks primarily in the 2θ range of 13–17°, indicating a highly crystalline structure. In Figure 4, CHS showed a small diffraction peak at approximately 2θ = 10° together with a broad diffraction peak within 2θ ≈ 15–26°, indicating the presence of limited ordered regions within a predominantly semi-crystalline structure. HEC exhibited a diffuse halo at 2θ ≈ 15–32°, characteristic of its amorphous nature. Poloxamer 188 displays sharp diffraction peaks at 2θ ≈ 19° and 23°, associated with crystalline polyethylene oxide domains, similar to PEG 20,000, confirming their high crystallinity.

**Figure 5:**
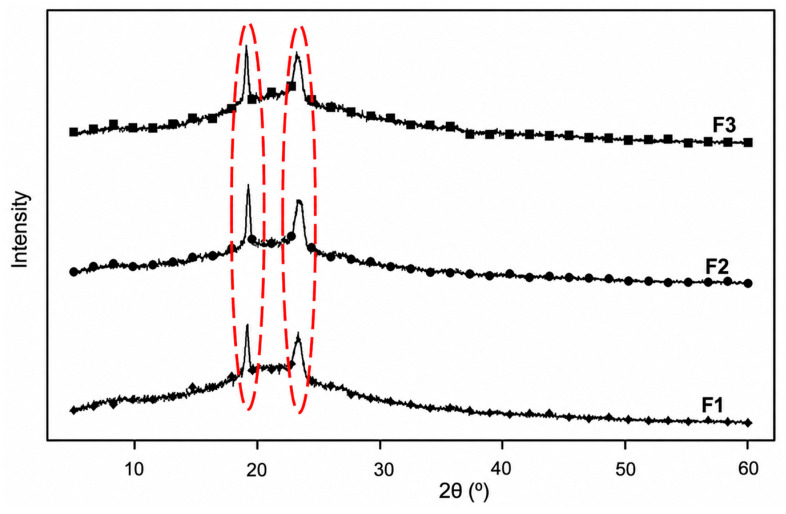
XRD patterns of the spray-dried microparticle formulations F1, F2 and F3. The red dashed circles indicate the characteristic diffraction peak positions used for visual comparison among F1–F3.

The XRD analyses of formulations F1–F3 as shown in Figure 5 indicate similar diffraction patterns, suggesting equivalent component ratios despite differences in spray-drying conditions. The formulations exhibited a semi-crystalline structure, characterized by diffraction peaks at 2θ ≈ 19° and 23°, due to Poloxamer 188. Broad regions were also observed, indicating the presence of amorphous components within the formulations.

**Figure 6:**
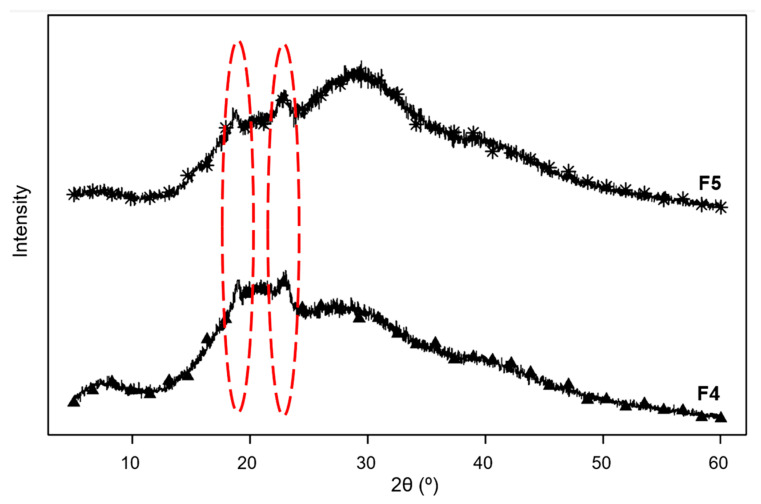
XRD patterns of the spray-dried microparticle formulations F4 and F5. The red dashed circles indicate the characteristic diffraction peak positions used for visual comparison among F4–F5.

In Figure 6, the XRD patterns of formulations F4 and F5 exhibited similar semi-crystalline characteristics, having major peaks at around 19° and 23°. These formulations exhibited wider diffraction peaks with an additional diffuse background at 2θ = 30°. The XRD profiles of all formulations also demonstrated an increasing broadening of peaks as particle size decreased from 3–8 µm (F1–F3) to 0.4–2 µm (F4 and F5).

The mechanical properties of the spray-dried formulations were studied in order to clarify the impact of formulation composition and internal structure on their mechanical behavior. The mechanical properties of the formulations are presented in Table 3.

The density values of all formulations were comparable and ranged from 1.26 to 1.29 g cm^−3^. The measured mechanical properties showed only minor variations among formulations. These data were used descriptively to support the interpretation of particle structure together with XRD and porosity results.

### 3.4. Encapsulation Efficiency of Andrographolide-Loaded Microparticles

**Figure 7:**
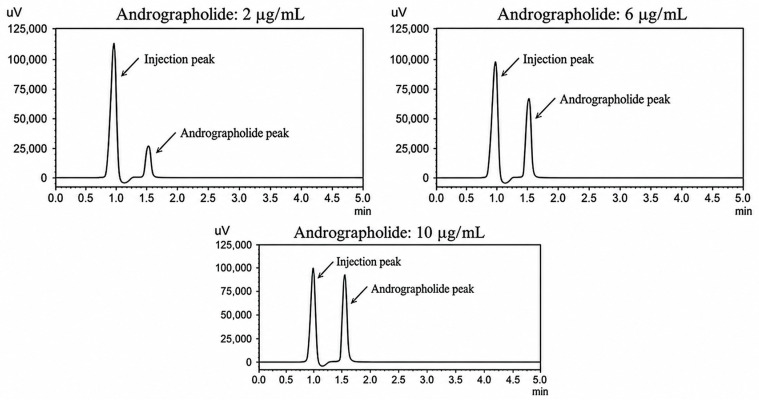
Representative HPLC chromatograms of pure andrographolide at various concentrations.

The encapsulation efficiency of andrographolide in the spray-dried microparticles was determined using HPLC. Representative chromatograms of pure andrographolide at concentrations of 2, 6, and 10 µg/mL are shown in Figure 7. The injection peak appeared at 1.1 min, while andrographolide was detected at a retention time of 1.55 min. The limit of detection (LOD) and limit of quantification (LOQ) were 0.35 µg/mL and 1.06 µg/mL, respectively. To assess the entrapment efficiency of the formulations, chromatograms of andrographolide extracted from formulations F1–F5 were analyzed and presented in Figure 7.

Table 4 summarizes the average andrographolide loading in the particles (per 5 mg) and the encapsulation efficiency. Formulations F1–F3 had identical component ratios; within this group, F2 and F3 showed comparable levels of both andrographolide loading and encapsulation efficiency, which were higher than those of F1. Among all formulations, F4 achieved the highest encapsulation efficiency (66.26%), while F5 showed the lowest (18.23%). Despite their different encapsulation efficiencies, F4 and F5 produced microparticles of comparable size (0.45–2 µm).

### 3.5. Encapsulation Efficiency and Release Behavior of Andrographolide

**Figure 8:**
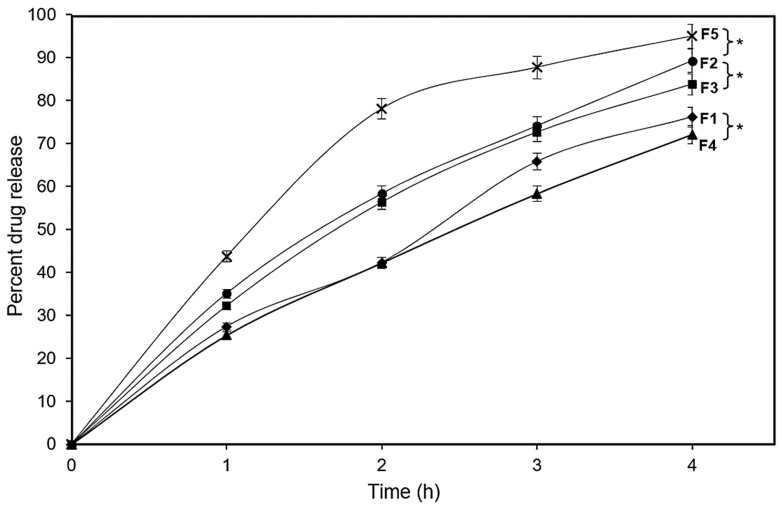
Cumulative percentage release of andrographolide from the spray-dried microparticle formulations F1–F5. * *p* < 0.05, as compared between two groups.

The cumulative percentage release of andrographolide from microparticle formulations F1 to F5 is presented in Figure 8. Formulation F5 exhibited the highest release rate during both the initial phase (0–2 h: 39.03%/h) and later phase (2–4 h: 8.46%/h), with a cumulative release of 94.7% ± 0.30% at 4 h. Among the formulations with comparable andrographolide levels (F2, F3, and F4), F2 and F3 showed significantly higher release rates (0–4 h: F2 = 22.31%/h; F3 = 20.94%/h; F4 = 18%/h; F2 compared to F4, *p* < 0.05; F3 compared to F4, *p* < 0.05) and cumulative release (at 4 h: F2 = 89.23% ± 0.18%; F3 = 83.76% ± 0.23%; F4 = 72.07% ± 0.20%; F2 compared to F4, *p* < 0.05; F3 compared to F4, *p* < 0.05).

### 3.6. Selection of the Optimal Formulation and Its Physicochemical Characterization and Antivirus Activity

**Figure 9:**
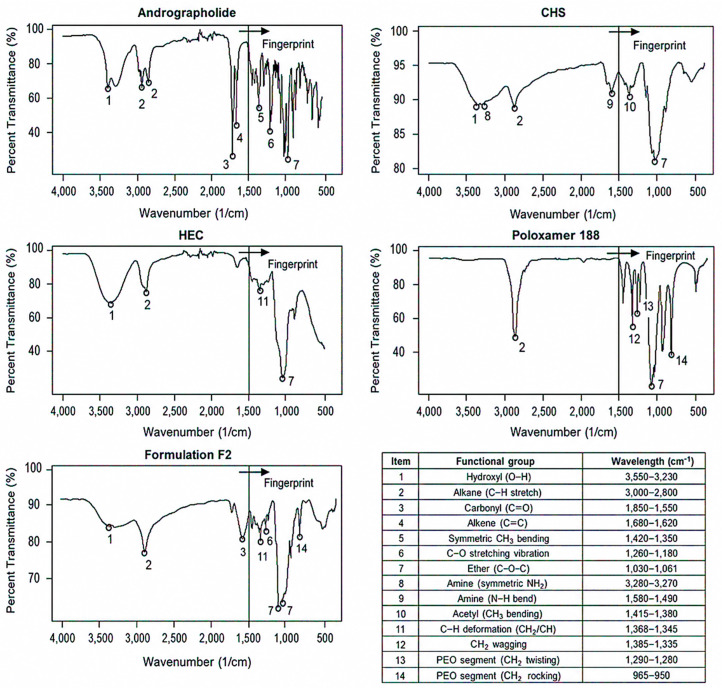
FTIR spectra of andrographolide, individual polymeric components, including chitosan (CHS; degree of deacetylation, 90%; MW, 150–400 kDa), hydroxyethyl cellulose (HEC; average MW, 90–250 kDa), Poloxamer 188 (MW, 7.6–9.5 kDa), and the spray-dried microparticle formulation F2.

**Figure 10:**
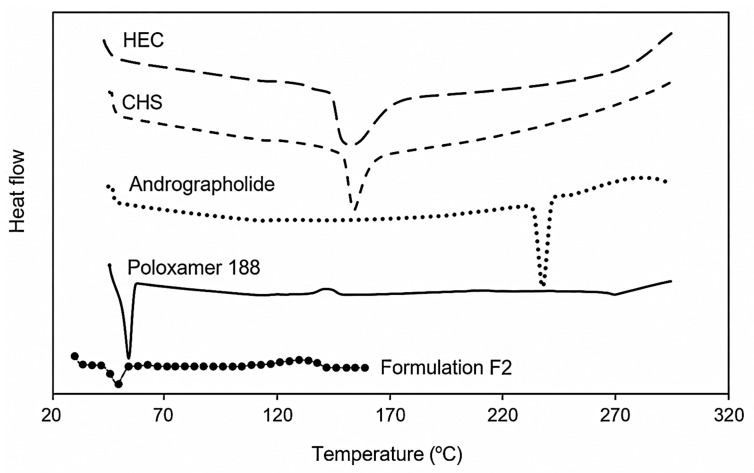
DSC thermograms of andrographolide, individual polymeric components, including chitosan (CHS; degree of deacetylation, 90%; MW, 150–400 kDa), hydroxyethyl cellulose (HEC; average MW, 90–250 kDa), Poloxamer 188 (MW, 7.6–9.5 kDa), and the spray-dried microparticle formulation F2.

**Figure 11:**
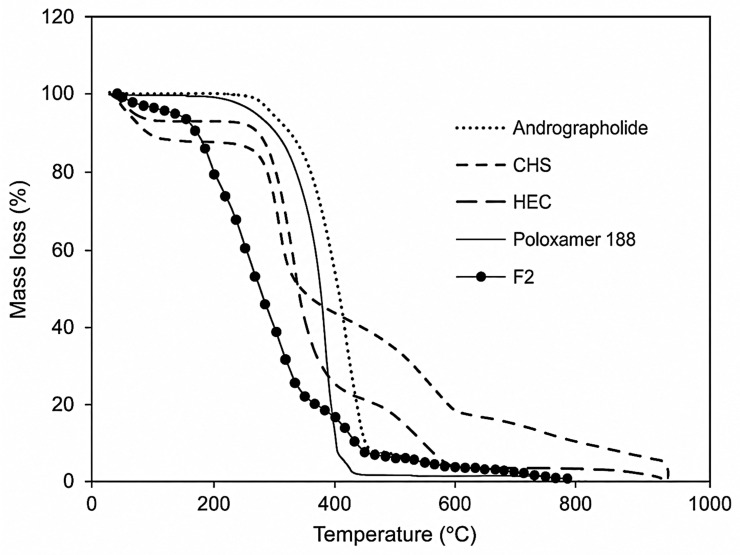
TGA thermograms of andrographolide, individual polymeric components, including chitosan (CHS; degree of deacetylation, 90%; MW, 150–400 kDa), hydroxyethyl cellulose (HEC; average MW, 90–250 kDa), Poloxamer 188 (MW, 7.6–9.5 kDa), and the spray-dried microparticle formulation F2.

The selection of the optimal formulation for andrographolide-loaded microparticles was based on the criteria explained in Section 2.6. Formulations F2 and F3 satisfied all the specified criteria. However, formulation F2 exhibited a higher cumulative release of andrographolide at 4 h than F3. Therefore, formulation F2 was selected as a suitable candidate for further investigations, including physicochemical characterization, followed by stability studies and antiviral activity evaluation. The chemical characteristics of andrographolide, polymer components, and microparticle formulation F2 were analyzed using FTIR (Figure 9), DSC (Figure 10), and TGA (Figure 11) to assess intermolecular interactions and thermal stability. Figure 9 indicates important functional groups characteristic of andrographolide, including alkene (C=C) stretching vibrations at ≈1680–1620 cm^−1^, as well as lactone-associated bands, namely the carbonyl (C=O) stretching vibration at ≈1850–1550 cm^−1^ and the corresponding C–O stretching vibrations at ≈1260–1180 cm^−1^. Furthermore, characteristic symmetric stretching vibrations of amine (–NH_2_) groups of CHS were observed at ≈3280–3270 cm^−1^, while the corresponding N–H bending vibrations appeared at ≈1580–1490 cm^−1^. HEC exhibited C–H deformation bands at ≈1348–1345 cm^−1^. The presence of poly(ethylene oxide) (PEO) from Poloxamer 188 was also confirmed. For formulation F2, the characteristic absorption bands of andrographolide, including carbonyl and C–O stretching vibrations, were retained, together with the polymeric signals, namely the amine (N–H bending) bands of chitosan, the C–H deformation bands of HEC, and the characteristic PEO bands of Poloxamer 188.

The thermal behavior of all components and formulation F2 was assessed to evaluate the compatibility and physical interactions among its constituent components. Figure 10 shows an endothermic peak for CHS and HEC at approximately 150 °C, corresponding to the glass-transition temperature (Tg) of their amorphous polymeric phases. Poloxamer 188 exhibited a distinct melting endotherm at 50–60 °C. The thermogram of formulation F2 displayed a similar melting event consistent with the melting point of Poloxamer 188, followed by a broad exothermic peak near 150 °C.

Further thermal characterization by TGA as shown in Figure 11 shows that CHS and HEC began to lose mass substantially between 250 and 270 °C, while andrographolide and Poloxamer 188 decomposed between 200 and 220 °C. Formulation F2 exhibited multiple weight-loss stages: the initial weight loss occurred between 180 and 200 °C, followed by a major decomposition stage at 380–400 °C.

### 3.7. Storage Stability of the Optimal Formulation

**Figure 12:**
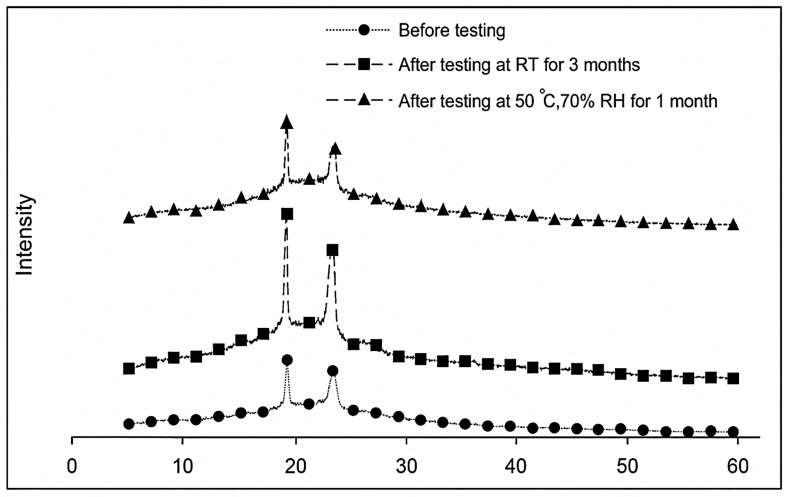
XRD pattern of the spray-dried microparticle formulation F2 before and after stability testing at room temperature (RT, 28 ± 2 °C, 50% RH) for 3 months and under accelerated thermal stress conditions (50 ± 2 °C, 70% RH) for 1 month.

The physicochemical properties of formulation F2 after storage under room temperature and accelerated thermal stress conditions were further evaluated using XRD and DSC, while the changes in andrographolide content following the stability study were quantitatively analyzed using HPLC. Figure 12 displays the XRD patterns of formulation F2 before and after storage under room temperature and accelerated thermal stress conditions, showing similar diffraction peak positions with characteristic peaks at approximately 18° and 23° (2θ).

**Figure 13:**
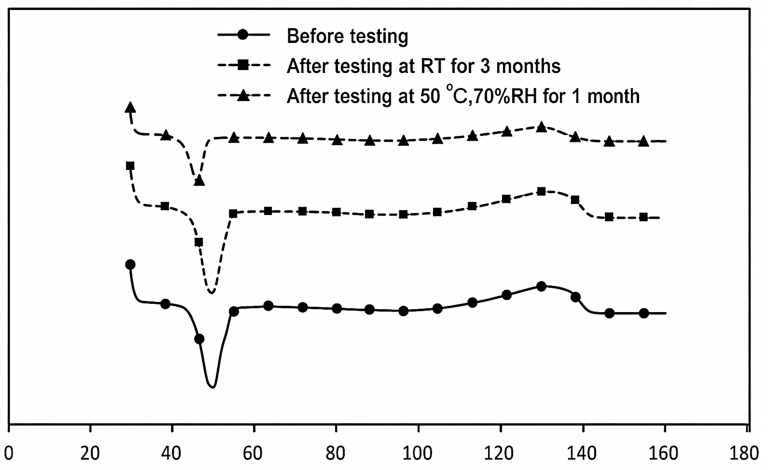
DSC thermograms of the spray-dried microparticle formulation F2 before and after stability testing at room temperature (RT, 28 ± 2 °C, 50% RH) for 3 months and under accelerated thermal stress conditions (50 ± 2 °C, 70% RH) for 1 month.

The DSC thermograms of the formulation F2 obtained after room temperature and accelerated thermal stress stability testing are shown in Figure 13. Under accelerated thermal stress storage at 50 ± 2 °C, 70% RH, the characteristic endothermic melting peak of Poloxamer 188 was retained with minimal change. A pronounced exothermic transition was also observed between 100 °C and 150 °C.

The chemical stability of andrographolide is summarized in Table 5. After 3 months of storage at room temperature, the andrographolide content decreased by only less than 1%, whereas accelerated thermal stress testing resulted in approximately 10% degradation.

### 3.8. Evaluation of Biological Activity of the Optimal Formulation

Antiviral efficacy was assessed based on log_10_ reduction in viral titer, as percentage reduction may mask differences at high levels of inactivation. Table 6 shows that the optimal formulation (F2) achieved a 3.3 log_10_ reduction, compared with 2.6 log_10_ for the control (placebo microparticles), indicating enhanced antiviral performance. A log_10_ reduction ≥ 3 is generally considered indicative of effective antiviral activity; therefore, formulation F2 met this criterion, whereas the control did not. These findings are consistent with a corresponding percentage reduction of 99.95% for formulation F2.

## 4. Discussion

This study presents the development of spray-dried microparticles encapsulating andrographolide. Five formulations (F1–F5) were developed and investigated to evaluate the influence of polymeric carrier compositions and spray-drying conditions on microparticle characteristics, including physicochemical and mechanical properties, encapsulation efficiency, release behavior, and biological activity.

Collective data from SEM analysis showed that formulations F1–F3, which had identical polymeric compositions, differed in particle size due to varying spray-drying conditions, while surface roughness remained comparable. Formulation F1, prepared at lower atomization air pressure (0.8 bar) and higher feed viscosity (20 cP), produced larger particles (6–8 µm). In contrast, formulations F2 and F3, processed at higher air pressure (1.1 bar) with lower viscosities (15 and 10 cP, respectively), yielded smaller particles (3–5 µm). These results confirm the influence of atomization air pressure and feed viscosity, as increased atomization pressure together with reduced feed solution viscosity led to smaller particle sizes in formulations F2 and F3. This behavior reflects the particle formation mechanism during spray drying, where droplet breakup and rapid solvent evaporation govern final particle size [45].

Formulations F4 and F5, which differed in polymeric composition, were prepared at higher atomization air pressure (1.5 bar) and lower feed viscosity (5 cP), producing substantially smaller particles (0.4–2 µm) with smoother surfaces but exhibiting agglomeration. The smooth morphology observed, even for inherently rough polymers such as CHS and HEC, is attributed to high atomization energy promoting uniform droplet breakup and rapid solvent evaporation, which limits polymer phase separation [46]. The reduced particle size was associated with lower surface charge due to a more compact polymer structure and decreased exposure of ionizable groups. In this submicron size range (<3 µm), reduced electrostatic repulsion allows van der Waals attractive forces to dominate, leading to particle agglomeration and poor dispersibility [28]. Simultaneously, submicron particles may remain suspended due to their low aerodynamic diameter and be readily exhaled, resulting in reduced pulmonary deposition efficiency [25]. A decrease in zeta potential indicates reduced electrostatic stabilization, with formulations F1–F3 showing values in the range of 3.75–4.50 mV, whereas formulations F4–F5 exhibited lower values of 2.25–2.75 mV. This reduction promotes particle agglomeration in aqueous dispersion due to weakened interparticle repulsion. In the dry state, the absence of a stabilizing medium leads to weaker permanent electrostatic binding, which may favor soft cohesion and enhance redispersion ability. In addition, the slightly positive surface charge may benefit pulmonary delivery by balancing penetration potential and mucoadhesion, resulting in optimized epithelial uptake while avoiding excessive mucus entrapment [15,25].

The XRD analysis revealed that all formulations exhibited semi-crystalline structures. For formulations F1–F3, the diffraction peaks were mainly attributed to Poloxamer 188, while no characteristic peaks of andrographolide were detected. This finding, together with FTIR results, indicated effective molecular dispersion of the andrographolide within the polymer matrix [23,31]. In contrast, F4 and F5 showed additional diffraction peaks corresponding to crystalline andrographolide, suggesting partial recrystallization within the polymer matrix. These formulations also exhibited reduced peak intensity and broader diffraction peaks in the 2θ range of 19–23°, reflecting decreased crystalline domain sizes and increased amorphous content. The reduced intensity of Poloxamer 188 peaks may be associated with a less ordered polymer arrangement, which could be influenced by higher atomization energy during spray drying [39].

Formulations F1–F3 exhibited slightly higher maximum and withdrawal forces together with marginally higher Young’s modulus values, indicating slightly greater stiffness and resistance to deformation, whereas formulations F4 and F5 showed slightly lower force and modulus values, reflecting reduced particle rigidity. This may be associated with the higher atomization air pressure, which promoted rapid solvent evaporation and less compact particle formation during spray drying. These mechanical trends are consistent with the XRD results, which revealed a partial reduction in crystallinity and changes in polymer chain organization in F4 and F5. These changes may have contributed to a slight decrease in stiffness [46]. Similar coefficient of restitution (COR) values (0.77–0.81) were consistent with comparable elastic recovery and collision behavior among the particles [47]. Collectively, the reduced mechanical strength observed in formulations F4 and F5 may be related to their higher porosity, resulting in a less compact particle structure, although this effect appears to have only a minor influence on elastic recovery behavior.

In summary, the results suggest that spray-drying conditions did not significantly affect the mechanical properties and therefore are not likely to be the dominant factor controlling formulation performance. Differences in polymer–drug composition and distribution within the matrix are more likely to contribute to variations in encapsulation efficiency and drug release. Nevertheless, parameters such as Young’s modulus, density, and COR remain important for describing particle interaction and transport behavior and can serve as useful inputs for future work on computational fluid–particle dynamics (CFPD) modeling to predict aerosol dispersion and deposition in the respiratory tract [48].

Regarding loading amount and encapsulation efficiency, formulations F1–F3, which had identical component ratios, exhibited moderate encapsulation efficiencies (approximately 30–50%). Within this group, formulations F2 and F3 showed higher andrographolide loading and encapsulation efficiency than formulation F1, indicating that spray-drying conditions, particularly atomization pressure and feed viscosity, influenced andrographolide retention under constant composition. This improvement in encapsulation efficiency can be attributed to the particle formation mechanism during spray drying, which resulted in enhanced drug entrapment within the polymer matrix.

In contrast, formulation F5, in the absence of PEG and containing a higher andrographolide loading, exhibited the lowest encapsulation efficiency (approximately 18%), indicating greater drug loss associated with weaker drug–polymer interactions, arising from reduced compatibility and increased surface localization of the drug due to preferential migration toward the particle surface during rapid solvent evaporation.

Regarding the release of andrographolide from the microparticles, formulation F5 exhibited the highest release rate and cumulative release. This behavior was associated with its high andrographolide loading and surface-localized drug distribution. These factors collectively promoted rapid drug release from the particles. Among formulations with comparable andrographolide content (F2–F4), formulation F2 showed the highest release efficiency, achieving approximately 89% cumulative release after 4 h. Formulations F1 and F3 exhibited slower release profiles than formulation F2, whereas formulation F4 displayed the slowest release behavior and the lowest cumulative release at 4 h. This may be attributed to the presence of PEG in formulation F4, which restricted drug mobility within the polymer matrix and limited drug availability at the particle surface, thereby retarding release.

Based on the overall performance, formulation F2 was selected as the optimal candidate for pulmonary administration. It met the key criteria outlined in Section 3.5: (1) particle size of 3–5 µm, (2) rough surface morphology with a zeta potential of 3.75 mV, (3) COR = 0.77 with Young’s modulus of 0.80 MPa, and (4) andrographolide encapsulation efficiency of 54.47% with a cumulative release of 89.23% after 4 h.

FTIR, DSC, and TGA results suggest that formulation F2 exhibited strong intermolecular interactions between andrographolide and the polymeric carriers (CHS, HEC, and Poloxamer 188), primarily mediated by hydrogen bonding and physical entrapment within the polymer matrix. The FTIR spectra showed characteristic functional groups of all components, including andrographolide, CHS amine groups, HEC C–H deformation bands, and Poloxamer 188-related PEO signatures, without the appearance of new peaks. This indicates the absence of chemical degradation or covalent modification, confirming physical incorporation of the drug within the matrix [49,50,51,52]. DSC analysis revealed a glass transition at approximately 150 °C corresponding to the amorphous CHS–HEC polymeric matrix, indicating increased molecular mobility of the polymer chains. In addition, a distinct melting endotherm observed at 50–60 °C attributed to Poloxamer 188, showed its retained crystalline thermal behavior within the formulation. TGA analysis confirmed that no significant mass loss occurred below 180 °C, indicating good thermal stability of the formulation under elevated temperature conditions relevant to the processing conditions evaluated in this study. The sequential thermal events observed in DSC prior to degradation further support that these transitions are associated with physical relaxation and phase behavior rather than chemical decomposition. Overall, these findings corroborate the applicability of formulation F2 as a spray-dried inhalable formulation, since it possessed a thermally stable semi-amorphous structure, in which andrographolide was molecularly dispersed within the CHS–HEC–Poloxamer 188 matrix.

The stability of formulation F2 was evaluated under room temperature and accelerated thermal stress storage conditions. XRD analysis showed no changes in diffraction patterns after storage, with characteristic peaks maintained at approximately 19° and 23° (2θ), indicating no detectable solid-state transformation or phase separation and preservation of the overall solid-state structure. DSC analysis further supported physical stability, as the melting endotherm of Poloxamer 188 was observed under both conditions. A broad endothermic transition between 100 °C and 150 °C was attributed to polymer relaxation and molecular rearrangement rather than degradation. It can be seen that the results of the XRD and DSC were consistent. HPLC results showed negligible loss of andrographolide under room temperature storage (>99% remaining after 3 months), whereas accelerated thermal stress conditions resulted in approximately 10% degradation, likely due to hydrolysis under elevated temperature and humidity.

The antiviral activity was expressed as a log_10_ reduction in viral titer. Formulation F2 achieved a 3.3 log_10_ reduction compared with 2.6 log_10_ for the placebo, meeting the ≥3 log_10_ criterion for satisfactory antiviral activity. This indicates retention of antiviral activity after spray drying, although the cell-free assay may not fully represent intracellular effects [53].

Overall, these results support the potential of polymer-based microparticles as an inhalable antiviral delivery system, with formulation F2 maintaining physicochemical stability and in vitro antiviral activity after spray drying. This work differs from previous andrographolide carrier systems studies because it combined an aqueous-based spray-drying process with a mixed-polymer strategy specifically designed for pulmonary delivery. In contrast, previous studies have applied single- or mixed-polymer systems, or solid-dispersion-based systems [21,22,23] using organic solvents, which may raise issues of pulmonary safety. Moreover, one of the studies [22] including another study which used a water-based system [24] focused on oral application. The optimal inhalable formulation developed in this study was proved for drug release, storage stability, and antiviral activity against Influenza A/H1N1. The aqueous-based process also reduces concerns related to residual organic solvents, scalability, and pulmonary safety. Further studies in cellular and in vivo systems, as well as computational fluid particle deposition (CFPD) modeling, are required to confirm the antiviral efficacy and pulmonary deposition performance of the formulation.

## 5. Conclusions

This study successfully developed and characterized spray-dried microparticles encapsulating andrographolide for potential pulmonary delivery applications. Five formulations were systematically evaluated to determine the optimal combination of polymer composition and processing parameters. The investigation revealed that both spray-drying conditions and formulation composition significantly influenced particle characteristics, with atomization air pressure and feed viscosity being critical factors controlling particle size distribution. Higher atomization pressures combined with lower feed viscosities promoted the formation of smaller particles through enhanced droplet breakup and rapid solvent evaporation.

Among all formulations tested, formulation F2 emerged as the optimal candidate, demonstrating superior overall performance across multiple evaluation criteria. This formulation achieved an ideal balance of particle size (3–5 µm), suitable for pulmonary deposition, moderate encapsulation efficiency (approximately 50%), and favorable release characteristics, achieving 89% cumulative andrographolide release within 4 h. The mechanical properties of formulation F2, including appropriate Young’s modulus value (0.80) and coefficient of restitution (0.77), indicated adequate particle integrity for inhalation delivery while maintaining efficient release.

Comprehensive characterization using FTIR, DSC, and TGA confirmed the thermal stability and molecular compatibility of formulation F2, revealing a semi-crystalline structure with andrographolide molecularly dispersed within the CHS–HEC–Poloxamer matrix through transient hydrogen bonding networks. The glass transition at approximately 150 °C and retained Poloxamer 188 melting endotherm indicated successful integration of all components without chemical modification. Stability studies demonstrated excellent physical and chemical stability under ambient conditions, with negligible andrographolide loss after three months at room temperature (>99% remaining). However, controlled storage conditions are recommended to minimize degradation under accelerated thermal stress storage conditions.

Most importantly, formulation F2 demonstrated significant antiviral efficacy against Influenza A/H1N1, achieving a 3.3-log_10_ reduction in viral titer, corresponding to approximately 99.95% inhibition. This finding confirms that andrographolide retained its biological activity following spray-drying encapsulation, validating the potential of this delivery system for respiratory antiviral therapy. The successful development of formulation F2 represents a promising approach for delivering andrographolide via inhalation, offering advantages in terms of targeted bioactive compound delivery, reduced systemic exposure, and enhanced therapeutic efficacy for respiratory viral infections.

## 6. Patents

A petty patent application was filed with the Thailand Department of Intellectual Property (DIP) on 23 April 2025, application number 2503001469, and is currently pending.

## Figures and Tables

**Table 1:** Formulations of andrographolide-loaded microparticles.

Formulation	Andrographolide (% *w*/*w*)	CHS (% *w*/*w*)	HEC (% *w*/*w*)	Poloxamer 188 (% *w*/*w*)	PEG 20,000 (% *w*/*w*)	Spray-Drying Parameter *		
						Solution Viscosity (cP)	Atomization Air Pressure (Bar)	Feed Rate (mL/min)
F1	0.6	62.2	15.5	21.7	–	20	0.8	6
F2	0.6	62.2	15.5	21.7	–	15	1.1	3
F3	0.6	62.2	15.5	21.7	–	10	1.1	3
F4	0.6	58	14.6	21.0	5.8	5	1.5	3
F5	5.8	58	9.6	26.6	–	5	1.5	3

* All spray-drying experiments were conducted at an inlet/outlet temperature of 120/90 °C.

**Table 2:** Physicochemical characteristics of the resulting spray-dried microparticles.

Formulation	Average Size (µm)	Surface Roughness	Zeta Potential (mV)	Estimated Surface Charge (V)
F1	6–8	Rough	4.50	0.36
F2	3–5	Rough	3.75	0.3
F3	3–5	Rough	3.75	0.3
F4	0.4–2	Smooth	2.75	0.22
F5	0.4–2	Smooth	2.25	0.18

**Table 3:** Mechanical properties of the spray-dried microparticle formulations.

Formulation	Bulk Density (g cm^−3^)	Maximum Force (N)	Withdrawal Force (N)	Young’s Modulus (MPa)	Coefficient of Restitution (COR)	Porosity
F1	1.26 ± 0.037	1.20 ± 0.018	0.95 ± 0.011	0.75 ± 0.012	0.79 ± 0.012	0.65 ± 0.010
F2	1.28 ± 0.038	1.30 ± 0.02	1.00 ± 0.012	0.80 ± 0011	0.77 ± 0.011	0.66 ± 0.010
F3	1.28 ± 0.038	1.30 ± 0.02	1.05 ± 0.012	0.82 ± 0.012	0.81 ± 0.012	0.66 ± 0.010
F4	1.29 ± 0.037	1.15 ± 0.017	0.92 ± 0.010	0.70 ± 0.010	0.80 ± 0.012	0.67 ± 0.011
F5	1.27 ± 0.038	1.10 ± 0.017	0.85 ± 0.010	0.65 ± 0.010	0.77 ± 0.011	0.68 ± 0.011

**Table 4:** Average andrographolide loading in microparticles and encapsulation efficiency.

Formulation	Average Amount of Andrographolide (µg/5 mg of Particles)	Encapsulation Efficiency (%)
F1	9.38 ± 0.05	31.27
F2	16.34 ± 0.10	54.47
F3	14.91 ± 0.09	49.70
F4	19.88 ± 0.10	66.26
F5	52.87 ± 0.29	18.23

**Table 5:** Andrographolide content in microparticle formulation F2 before and after stability testing.

Condition	Andrographolide Content (µg/5 mg Particles)	Andrographolide Remaining (%)
Before testing	16.34 ± 0.09	100.00
Room temperature (3 months)	16.27 ± 0.05	99.57
Accelerated thermal stress (50 °C, 70% RH) 1 month)	14.65 ± 0.10	89.65

**Table 6:** Antiviral activity against influenza A/H1N1 of the optimal formulation.

Sample	Exposure Time	Titer of Virus Recovery Control ${TCID}_{50}/100\;\mu L$	Titer of Virus After Disinfection by the Test Product ${TCID}_{50}/100\;\mu L$	Log_10_ Reduction of Virus Titer on Average
Placebo	60 min	$10^{7.1}$	$10^{4.5}$	2.6
Formulation F2	60 min	$10^{7.1}$	$10^{3.8}$	3.3

## Data Availability

Data are contained within the article.

## Supplementary Materials

The following supporting information can be downloaded at: https://www.mdpi.com/article/10.3390/polym18131655/s1, Figure S1: Representative SEM image of microparticles from preliminary spray-drying feasibility screening; Table S1: Qualitative screening of Poloxamer 188 concentration range for 10 mg andrographolide dispersion; Table S2: Preliminary screening of CHS–HEC ratios based on visual dispersion stability; Table S3: Physicochemical and functional properties of polymeric carriers used [16]; Table S4: Physicochemical properties of solubility agent; Figure S2: XRD patterns of the spray-dried microparticle formulations F1, F2, and F3; Figure S3: XRD patterns of the spray-dried microparticle formulations F2, F4, and F5; Figure S4: Representative HPLC chromatogram of pure andrographolide at various concentrations during 0.8–1.8 min; Figure S5: HPLC chromatograms of andrographolide extracted from the spray-dried microparticle formulations F1–F5 during 0.8–1.8 min; Figure S6: DSC thermograms of andrographolide, individual polymeric components, and the spray-dried microparticle formulation F2. References [54,55] are cited in the Supplementary Materials.
