# Supplementary material for: Development and Characterization of Andrographolide Microparticles via Spray Drying: An Aqueous-Based Chitosan/Cellulose/Poloxamer Carrier Approach
Source: Polymers (Basel). 2026 Jul 3;18(13):1655. doi: 10.3390/polym18131655 (PMC13364418; doi:10.3390/polym18131655)
Supplement: Supplementary file 1 [file polymers-18-01655-s001.zip › polymers-4340071-supplementary.pdf]

Supplementary Information

Development and Characterization of Andrographolide Mi-croparticles via Spray Drying: An Aqueous-Based Chi-tosan/Cellulose/Poloxamer Carrier Approach

Nuttapong Khiaonoi 1, Kwanchai Kraitong 1, Punyawan Lumpaopong 1,* and Jarupa Viyoch 2,*

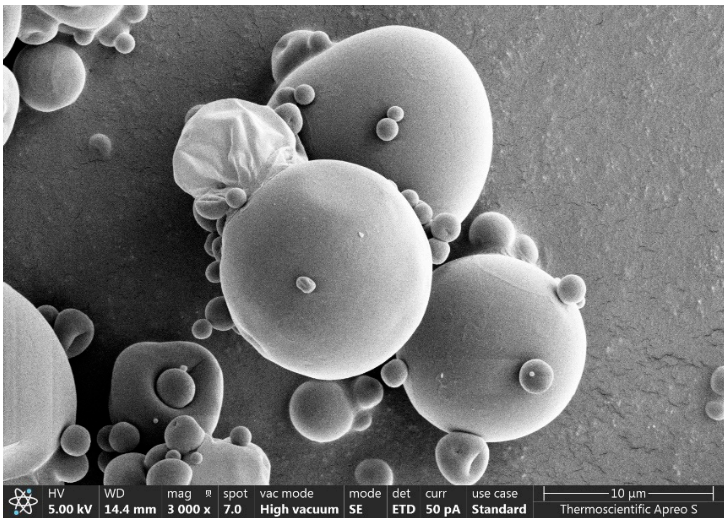

Figure S1. Representative SEM image of microparticles from preliminary spray-drying screening.

Table S1. Qualitative screening of Poloxamer 188 concentration range for 10 mg andrographolide dispersion.

Trial	Poloxamer 188 Concentration	Visual appearance after mixing	Visible andrographolide precipitation
**P1**	0.50 ± 0.17% w/v	non-homogeneous dispersion	Observed
**P2**	1.00 ± 0.33% w/v	non-homogeneous dispersion	Slightly observed
**P3**	3.00 ± 1.00% w/v	homogeneous dispersion	Not observed

Table S2. Preliminary screening of CHS:HEC ratios based on visual dispersion stability.

CHS:HEC ratio	CHS Concentration	HEC Concentration	Visible polymer aggregation
**2:1**	1 % w/v	0.5% w/v	Observed
**3:1**	1 % w/v	0.3% w/v	Observed
**4:1**	1 % w/v	0.25% w/v	Not observed

Table S3. Physicochemical and functional properties of polymeric carriers used [16]

Polymer	Surface Charge (in aqueous systems)	Surface Morphology	Mucus Adhesion /Penetration	Enhancement of Therapeutic Effect
**CHS**	Positive	Highly rough	Mucus adhesion	Absorption and bioavailability enhancement
**HEC**	slightly negative apparent	Moderately rough	Moderate mucus permeability	Dispersibility enhancement
**PEG 20,000†**	Neutral	Smooth	Mucus permeability	Solubilizer and Stabilizer

† PEG 20000 is included in this table because it contributes to polymer-related \ characteristics.

Table S4. Physicochemical properties of solubility agent

Solubility Agent	Critical Micelle Concentration	Hydrophilic–Lipophilic Balance (HLB)	Kinematic Viscosity (cP)	Solubility of Andrographolide
**Poloxamer 188 [54]**	≈20–40 mg/L	≈29	160–400*	Moderately soluble*
**PEG 20,000 [55]**	Not applicable	≈18–20†	140–180*	Slightly soluble*

* Viscosities were measured using a viscometer at ≈5–10% (w/v) Poloxamer 188 and ≈3–5% (w/v)
PEG 20,000. Solubility was qualitatively assessed based on visual observation of dispersion.
† The HLB value for PEG 20,000 represents an estimate based on Griffin's method.

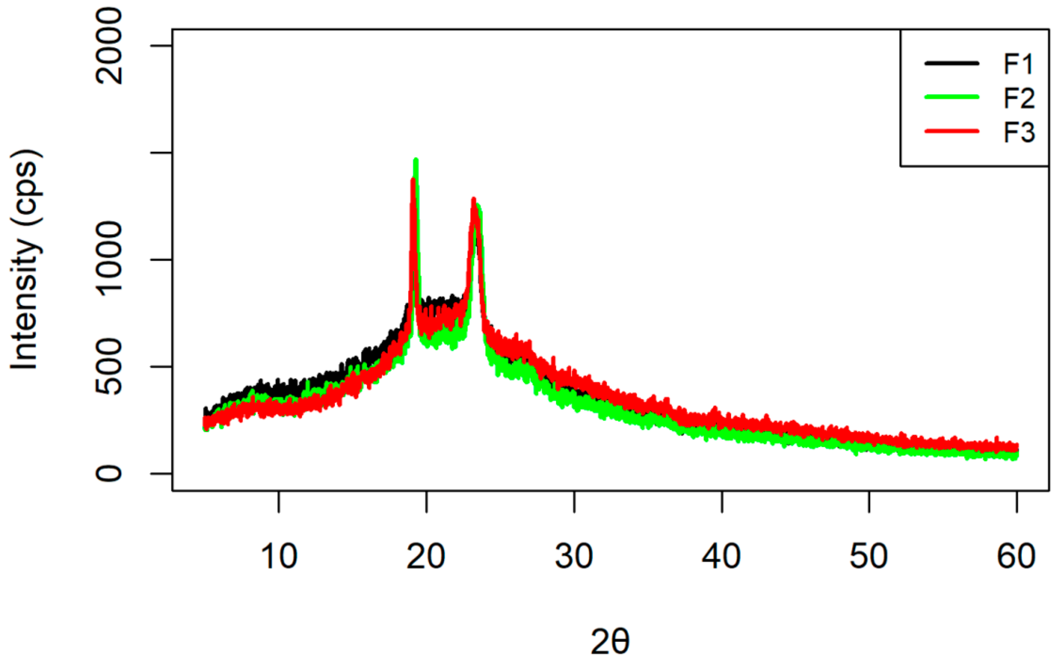

Figure S2. XRD patterns of the spray-dried microparticle formulations F1, F2, and F3.

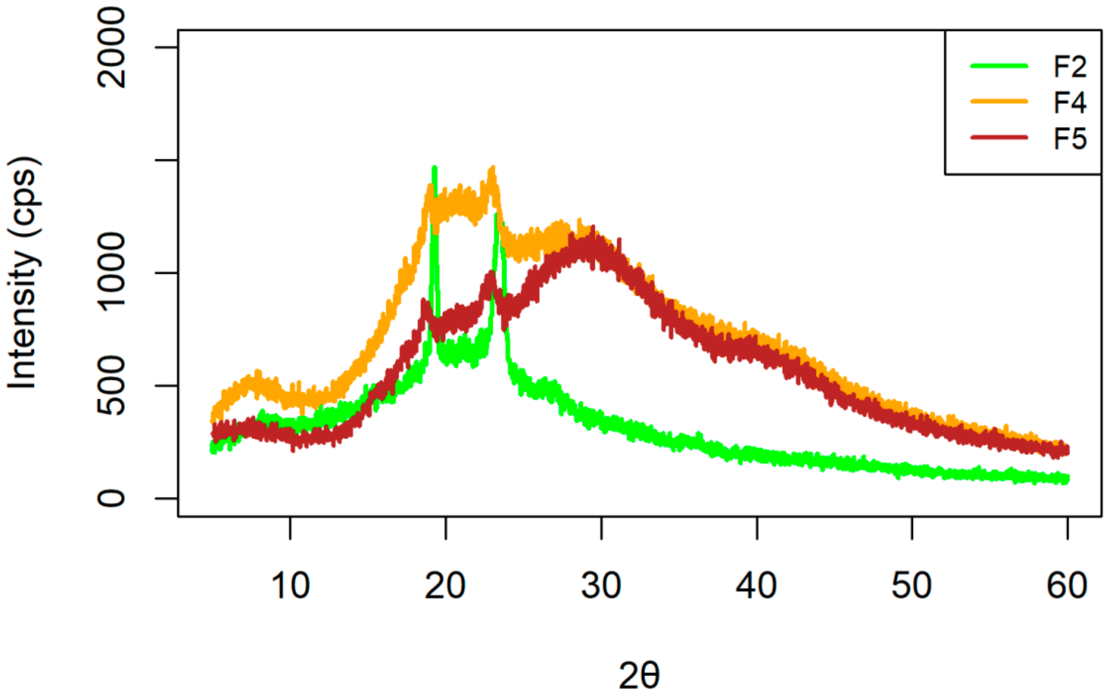

Figure S3. XRD patterns of the spray-dried microparticle formulations F2, F4, and F5.

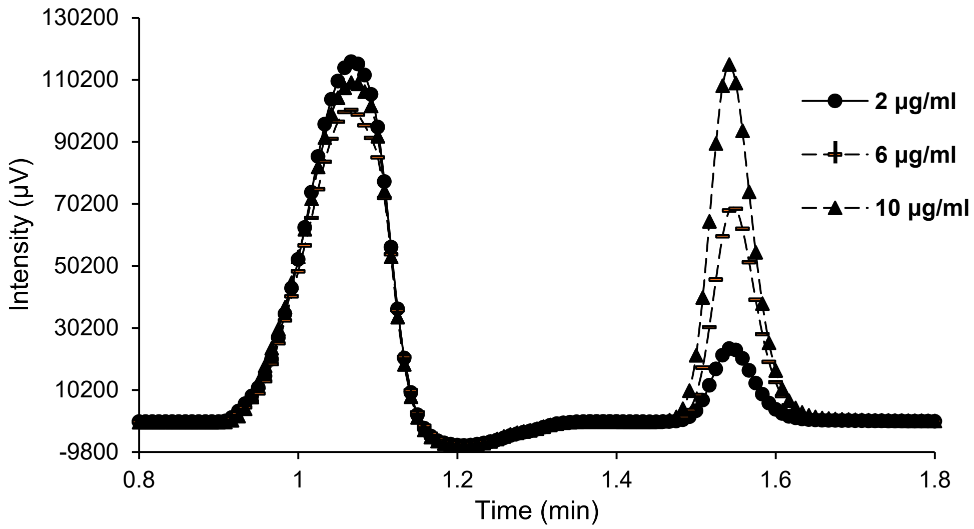

Figure S4. Representative HPLC chromatogram of pure andrographolide at various concentrations during 0.8-1.8 min.

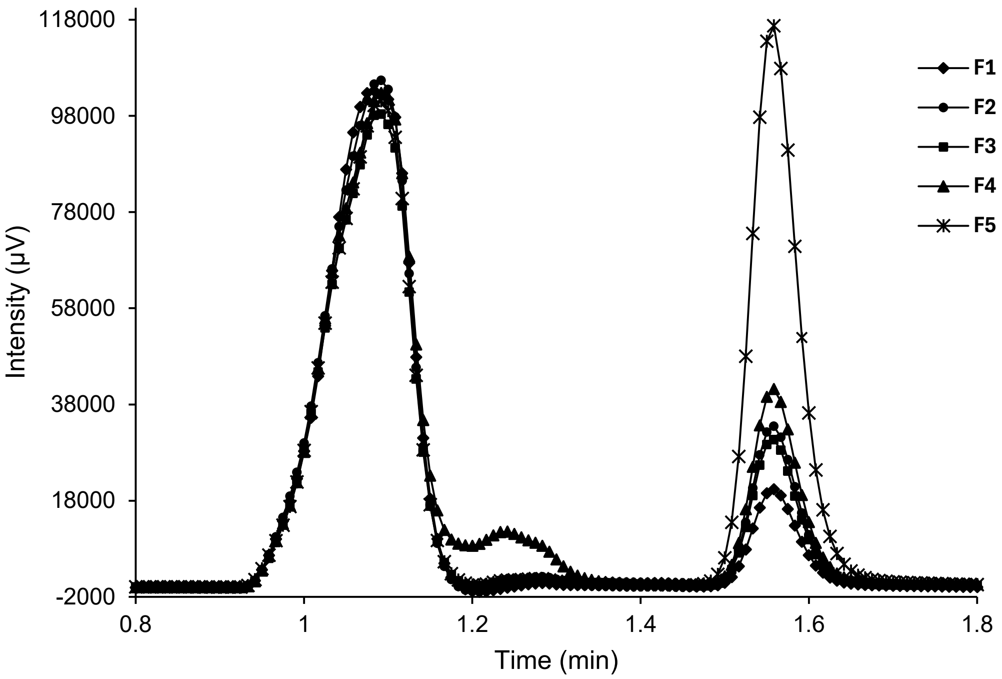

Figure S5. HPLC chromatograms of andrographolide extracted from the spray-dried microparticle formulations F1–F5 during 0.8-1.8 min.

.

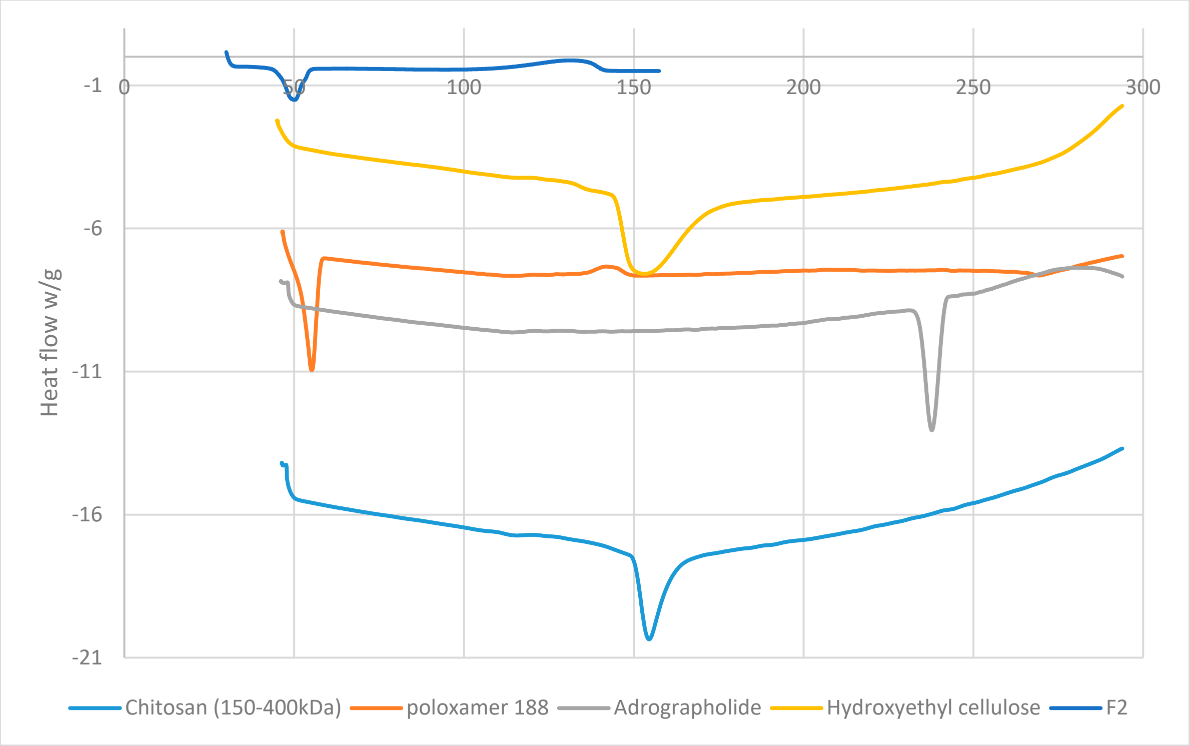

Figure S6. Multiple plot of DSC thermograms of andrographolide, individual polymeric components and the spray-dried microparticle formulation F2.
